# Organokines and Exosomes: Integrators of Adipose Tissue Macrophage Polarization and Recruitment in Obesity

**DOI:** 10.3389/fendo.2022.839849

**Published:** 2022-02-18

**Authors:** Yuan-Yuan Wang, Ya-Di Wang, Xiao-Yan Qi, Zhe-Zhen Liao, Yun-Ni Mai, Xin-Hua Xiao

**Affiliations:** The First Affiliated Hospital, Department of Metabolism and Endocrinology, Hengyang Medical School, University of South China, Hengyang, China

**Keywords:** obesity, adipose tissue inflammation, adipose tissue macrophages, organokines, exosomes

## Abstract

The prevalence of obesity is escalating and has become a worldwide health challenge coinciding with the development of metabolic diseases. Emerging evidence has shown that obesity is accompanied by the infiltration of macrophages into adipose tissue, contributing to a state of low-grade chronic inflammation and dysregulated metabolism. Moreover, in the state of obesity, the phenotype of adipose tissue macrophages switches from the M2 polarized state to the M1 state, thereby contributing to chronic inflammation. Notably, multiple metabolic organs (adipose tissue, gut, skeletal muscle, and the liver) communicate with adipose tissue macrophages *via* secreting organokines or exosomes. In this review, we systematically summarize how the organokines (adipokines, gut microbiota and its metabolites, gut cytokines, myokines, and hepatokines) and exosomes (adipocyte-, skeletal muscle-, and hepatocyte-derived exosomes) act as important triggers for macrophage recruitment in adipose tissue and adipose tissue macrophage polarization, thus providing further insight into obesity treatment. In addition, we also highlight the complex interaction of organokines with organokines and organokines with exosomes, revealing new paths in understanding adipose tissue macrophage recruitment and polarization.

## 1 Introduction

As per the World Health Organization, since the mid-1970s, the global prevalence of obesity has tripled, with an estimated 1 billion adults being overweight, as well as 650 million adults and 124 million children and adolescents being obese. Obesity escalates the risk of acquiring diverse diseases for instance diabetes mellitus (1), cardiovascular diseases (1), certain types of cancer (2), a range of musculoskeletal conditions (3), and poor mental health (4). This results in a huge financial burden on public healthcare costs worldwide. Therefore, it is imperative to improve our comprehension of the mechanisms accounting for obesity’s pathogenesis.

Obesity is defined as a chronic imbalance of caloric intake with energy expenditure. Moreover, it has been recognized as a low-grade, chronic inflammatory condition that is followed by metabolic dysfunction along with insulin resistance (IR) (5). Even though the molecular basis underlying the inflammation is not yet fully understood, researchers agree that adipose tissue macrophages (ATMs) contribute to a pro-inflammatory state in obesity and obesity-associated metabolic dysfunctions (6, 7). In an obese state, a series of chemokines, monocyte chemotactic protein-1 (MCP-1), and leukotriene B4 (LTB4), attract monocytes into the adipose tissue. These recruited monocytes are termed ATMs and are generally accompanied by a phenotypic switch (most commonly M1/M2), also known as ATM polarization (8, 9). Compared to lean mice and humans, there is an increase in macrophage infiltration into adipose tissue in obesity (6). This is because of the influx of bone marrow–originated precursors into the adipose tissue along with their subsequent differentiation to generate mature F4/80-expressing macrophages (6). More importantly, ATMs from lean mice express elevated levels of genes for instance Ym1, arginase 1 (Arg1), and IL-10, which are traditionally linked to ‘alternatively activated’ or M2-like macrophages. On the other hand, ATMs originated from obese mice express elevated levels of genes for instance tumor necrosis factor alpha (TNF-α) and inducible nitric oxide synthase (iNOS), which are characteristic of ‘classically activated’ or M1-like macrophages (9). In addition, both ATM populations can be distinguished based on the expression of surface markers, where most ATMs express CD206 in lean mice, whereas in obese conditions, CD11c is upregulated (9). Except for the classic M1 and M2 phenotypes, newly identified subpopulations of macrophages (e.g. MMe, LAMs, VAMs, and MFehi ATMs) are related to multiple metabolic regulators (10–13). However, in the coming years, it will be important to understand the precise role of these newly identified phenotypes in obesity.

**Figure 1:**
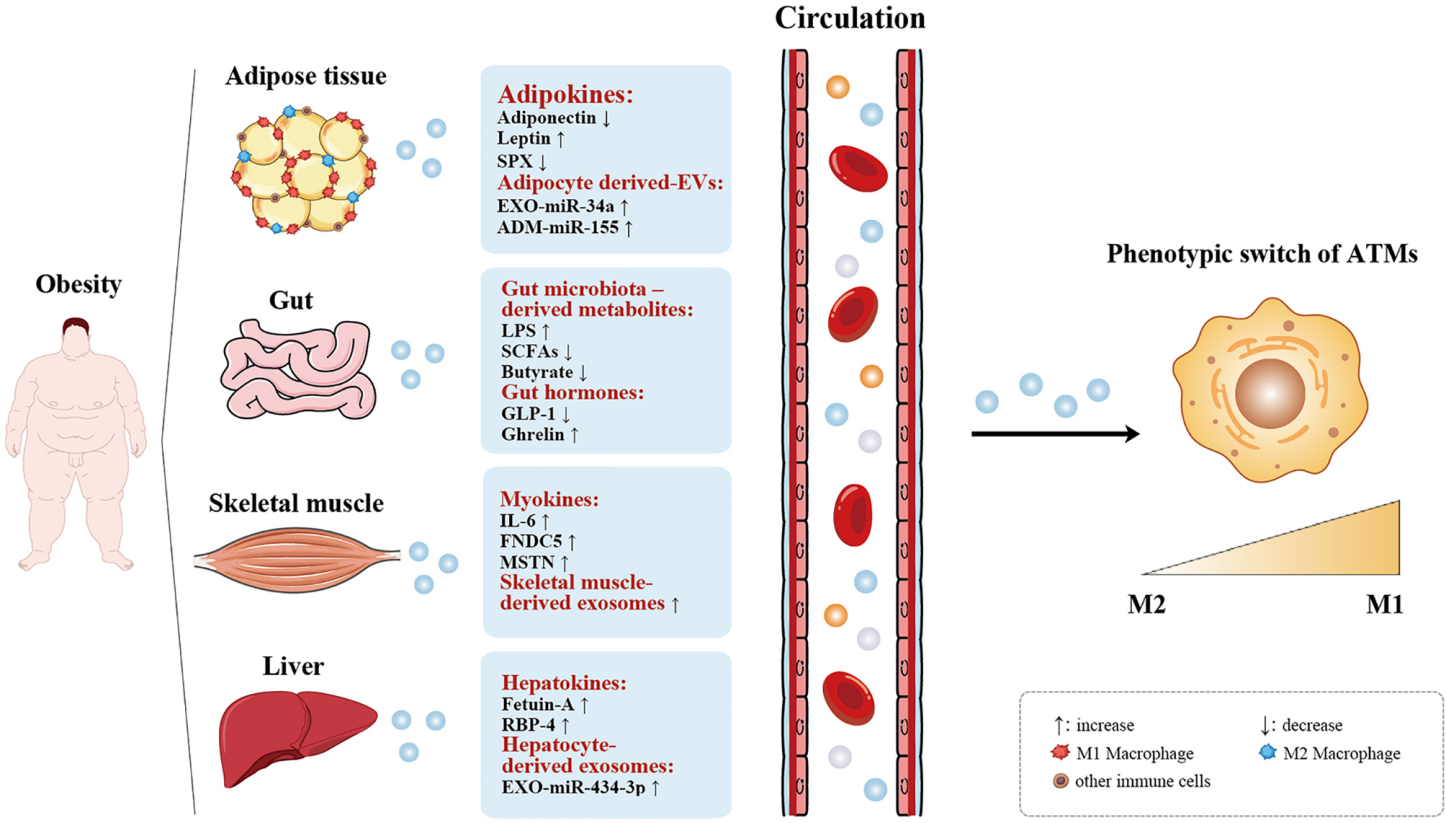
Organokines and exosomes-mediated ATM polarization. Adipose tissue, gut, skeletal muscle, and the liver act as endocrine organs by secreting organokines (adipokines, gut microbiota-derived metabolites, gut hormones, myokines, and hepatokines) and exoxomes (adipocyte-, skeletal muscle-, and hepatocyte-derived exosomes), which are increased or decreased in obesity, thus affecting the phenotype of ATMs switch from the anti-infammatory (M2) state to the pro-infammatory (M1) state. ↑ increase, ↓ decrease.

Adipose tissue, the liver along with the skeletal muscle are the main metabolic organs that are involved in obesity-associated metabolic disturbances *via* biosynthesis coupled with secretion of adipokines, myokines, as well as hepatokines, respectively (14). It is worth noting that these metabolic organs also secrete exosomes to communicate with peripheral cells along with distant organs and modulate whole-body metabolism. In recent years, growing research evidence has demonstrated that the gut plays a crucial role in the onset and development of many metabolic diseases *via* secretion of gut hormones, the gut microbiome, and its metabolites (which can also be defined as an organokines) (15, 16). Here, we summarize organokines such as adipokines, gut microbiota and its metabolites, gut hormones, myokines, and hepatokines, that are responsible for recruiting macrophages and shifting the ATM phenotype, thus initiating inflammatory cascades (**Figure 1**). In addition, we focused on the emerging role of exosomes produced by these endocrine organs on the polarization of ATMs (**Figure 1**). Of note, we also emphasize these organokines and exosomes can interact and regulate each other, talking indirectly to ATMs. With increasing understanding of organokines and exosomes in regulating ATM recruitment and polarization, novel insights, as well as treatment strategies should emerge in the prevention of obesity.

## 2 The Crosstalk Between Adipose Tissue and ATMs

Adipose tissue is the primary fat storing tissue and is also recognized as the largest endocrine organ secreting adipokines systematically (17). In the setting of obesity, adipokines elicit recruitment of immune cells (mostly macrophages). Moreover, they play indispensable roles in the crosstalk of adipocytes with macrophages, thus modulating AT inflammation (17). More recently, it has been proposed that adipocyte-derived exosomes are involved in adipocyte/macrophage crosstalk and act as a remarkable mediator, whereby adipocytes modulate the polarization of ATMs in obesity (18, 19). Many research studies have aimed to elucidate the mechanisms *via* which adipocytes interact with macrophages and contribute to adipose tissue inflammation in obesity (20). Here, we summarize adipokines and adipocyte-derived exosomes, which are all important for macrophage recruitment and ATM activation in an obese state, thus regulating adipose tissue inflammation.

### 2.1 Adipokines

To date, many adipokines have been identified to modulate adipose tissue inflammation by switching the macrophage phenotype or accumulation of ATMs (21–23). The effect of some well-documented adipokines or lesser-known adipokines on ATMs polarization and recruitment in obesity are summarized in a chart (**Supplementary Table 1**). In this section, we describe in detail the role and mechanisms of three classical and newly identified adipokines (adiponectin, leptin, and SPX).

#### 2.1.1 Adiponectin

Adiponectin, the most abundant adipokine produced by adipose tissue, is downregulated in obesity and has a negative relationship with chronic inflammatory states; it is implicated in the immune response *via* modulating macrophage proliferation along with polarization. Cell culture investigations have documented that adiponectin influences the role of macrophages in several ways, consisting of the repression of the expressions of class A scavenger receptor (24), diminished nuclear factor-kappaB (NF-κB) activation *via* the Toll-like receptors (TLRs) (25), and upregulation of the anti-inflammatory cytokine IL-10 (26). Furthermore, Ohashi K et al. reported that ATMs originated from adiponectin-deficient mice display activation of M1 markers and suppression of M2 markers. In a cell culture, adding recombinant adiponectin to macrophages results in an upregulation of M2 markers, which was found to also reduce the level of M1 biologic signatures and trigger the expression of M2 biologic signatures in human monocyte-originated macrophages, as well as stromal vascular fraction (SVF) cells purified from human adipose tissue (27). As early as 2015, Hui et al. have demonstrated that adiponectin is relevant for cold-induced browning of subcutaneous white adipose tissue *via* promotion of M2 macrophage proliferation (28). However, other studies have demonstrated adiponectin as a negative regulatory factor of cold-induced browning/thermogenesis (29–32). One possible speculation for these conflicted results is that the effect of adiponectin on thermogenesis may be mediated simultaneously through multiple adiponectin receptors or adiponectin signaling (29). Thus, future studies need to clarify these confounding results and the underlying mechanisms of adiponectin in the regulation of energy. Overall, adiponectin directly regulates the macrophage phenotype, leading to the switch from a proinflammatory M1-like state to an anti-inflammatory M2-like state.

#### 2.1.2 Leptin

Obesity is linked to an elevated content of leptin in the expanding adipose tissue (33), and hyperleptinemia could result into low-grade systemic inflammation in obesity, illustrating a possible function of leptin in obesity-triggered inflammation (34). Dib et al. reported that C57BLJ mice reconstituted with db/db (leptin receptor-deficient) bone marrow, when fed on a high-fat diet (HFD), have a remarkably reduced body weight and adiposity, alleviated macrophage infiltration, and successively decreased adipose tissue inflammation, as well as a switch of ATMs from proinflammatory (M1) to anti-inflammatory (M2) *in vivo* (35). Importantly, two recent studies further supported that a reduction in leptin levels in an obese state serves as a novel strategy for loss of weight coupled with insulin sensitization (36, 37). Zhao et al. generated two diverse models of mouse of partial leptin insufficiency: an adipocyte-distinct congenital heterozygous leptin knockout mouse line (LepHZ) and a well-developed whole-body heterozygous leptin knockout mouse (OBHZ). In response to HFD, OBHZ and LepHZ mice were resistant to diet-triggered obesity, driven mostly *via* decreased fat mass expansion with no effect on lean mass. Moreover, macrophage infiltration into adipose tissue was greatly reduced in the epididymal adipose tissue depot of OBHZ and LepHZ mice on an HFD, accompanied by a reduction in adipose tissue inflammation markers (Mcp1, F4/80, and TNF-α) (36). Thus, partially leptin deficient mice under obesogenic conditions display reduced ATM recruitment, leading to a healthy adipose tissue phenotype. However, on chow fed diet, male OBHZ and LepHZ mice showed increased body weights (36), consistent with the classical physiological role attributed to leptin. The two different settings, i.e. leptin-sensitive (chow fed diet) and leptin-resistant (high fed diet) states can explain this contrary result (36, 37). In the leptin-sensitive state, increasing leptin levels did reduce food intake and body weight, while decreasing leptin levels resulted in increased food intake and body weight. In contrast, in the obese state, partial deficiency of leptin restored leptin sensitivity, resulting in a decrease in food intake and weight loss, accompanied by improved insulin sensitivity. Because secretion of catecholamines by anti-inflammatory macrophages induces adipose tissue uncoupling protein 1 (UCP1) (38), we speculated whether the reduction in proinflammatory M1 phenotypes in OBHZ and LepHZ mice on HFD plays a similar role, thus explaining the difference in body weight. Although they did not measure the expression of UCP1 in these two mouse models, we believe that this hypothesis can be explored in the future.

#### 2.1.3 Spexin (SPX)

SPX, a 14-amino-acid peptide, constitutes a novel adipokine, which was first predicted *via* bioinformatics tools in 2007 (39). In diet-induced obese (DIO) mice, a low SPX plasma level, as well as low adipose tissue content were seen, and upon treatment with SPX, there was a decrease in caloric intake and body weight loss accompanied with improved glucose tolerance test (40). Evidence suggested that SPX dampens the uptake of fatty acid *via* adipocytes and that it represses the adipogenic process in the 3T3-L1 preadipocyte cell line (40, 41). Recently, a study evaluated the influence of SPX treatment on adipose tissue inflammation and ATM populations in a previously fructose-rich-diet (FRD) obese mouse model. This obese mouse model was characterized by an increase in body weight and epididymal adipose tissue masses, and an enhancement of inflammation in adipose tissue correlated with a decrease in M2 and an increase in M1 ATMs (42). SPX was found to reduce the percentage of M1 macrophages and increase M2 ATMs, and to improve adipose tissue inflammation, metabolic profile and adipocyte hypertrophy during FRD induced obesity (43).Furthermore, these data reported *in vivo* could be, at least partly, because of the direct action of SPX on macrophage activation. Thus, these data illustrate that SPX exerts a beneficial effect in inhabiting adipose tissue inflammation during obesity with a possible therapeutic potential for treating obesity.

### 2.2 Adipocyte-Derived Exosomes

Extracellular vesicles (EVs consisting of exosomes, microvesicles, and apoptotic bodies), a new class of microscopic endocrine hormone, mediate intercellular communication at the molecular level (44). Exosomes are nano-sized membrane-bound vesicles containing RNA (mRNA, miRNA, as well as other RNAs), protein, and lipids, many of which have gained much research attention for their roles in metabolic disorders, specifically in obesity along with its complications (44). Exosomes are generated from adipocytes and have been suggested to participate in adipocyte/macrophage crosstalk (18). Importantly, adipocyte-derived exosomes have also been found to control the ATM phenotype and play key roles in modulating adipose tissue inflammation.

Investigations have documented that adipose tissue might be a primary source of circulating exosomal miRNAs, which modulate metabolic homeostasis, as well as directly enhance IR in other organs (19). For example, a growing body of evidence has demonstrated that obese rodents and humans have greater levels of adipocyte-secreted exosomal miR-34a, and this correlates well with IR and metabolic inflammation (45–47). Importantly, Pan showed that miR-34a in adipocyte-derived exosomes suppress M2 polarization by targeting the transcription factor Kruppel-like factor 4, which in turn induces metabolic inflammation and IR. In this study, they also verified that adipose-selective ablation of miR-34a mice led to an obvious reduction in the adipose-resident M1 macrophages as well as significant upregulation of the M2 macrophages, which is accompanied by lower protein levels of the M1 macrophage marker (iNOS), coupled with an elevated abundance of the M2 macrophage marker Arg1 (47). Likewise, Zhang et al. showed that miR-155 in adipocyte-derived microvesicles could regulate macrophage polarization, hence eliciting the M2-to-M1 switch and inflammation *via* Janus kinase (JAK)/signal transduction and activator of transcription (STAT) signaling (48). Melatonin administration increased adipose-derived exosomal α-ketoglutarate (αKG), which was subsequently transported to macrophages; then, M2 macrophage activation was promoted, verifying the crosstalk between adipocytes and macrophages (49). Collectively, these data indicate that adipocyte-derived exosomes can regulate ATM polarization in a paracrine fashion, revealing a novel mechanism of obesity-induced chronic inflammation.

## 3 Crosstalk Between the Gut and ATMs

Evidence documents that the gut is a key site that is altered in obesity and obesity-linked inflammation. These alterations constitute changes in gut hormones, gut microflora, and their metabolites, which act as possible triggers of inflammation in obesity (50, 51). Among these gut-derived factors related to obesity, we will summarize existing knowledge on the potential of gut microbiota and its metabolites (including LPS, SCFAs, and butyrate), and gut hormones (GLP-1, Ghrelin) directly or indirectly modulate the activation of ATMs and mediate the pathogenesis of obesity-related inflammation.

### 3.1 Gut Microbiota-Derived Metabolites

Recently, emerging research evidences have shown that the gut microflora and its metabolites play critical roles in the onset, as well as development of obesity, and that the gut microbiota has lower bacterial richness in obese patients, along with increased IR and a markedly inflammatory phenotype (16, 52). In addition, the gut microbiota proportion was also altered in obesity, while obese people had a higher Firmicutes and lower Bacteroidetes proportion compared to lean people (50). Crosstalk between the gut microbiome and ATMs was initially observed in germ-free mice and ameliorated adiposity and resistance to obesity progression when they were fed an HFD. Caesar et al. also demonstrated that colonization of germ-free mice with Escherichia coli could result in the aggregation of macrophages in WAT and drive a shift of WAT macrophages from the anti-inflammatory M2 phenotype toward the proinflammatory M1 phenotype (53). While the role of the gut microbiome in modulating the ATM phenotype is in its early stage, more studies are needed to elaborate the complex interplay between them.

The gut microbiota produces a variety of metabolites, including lipopolysaccharide (LPS), short-chain fatty acids (SCFAs), bile acids, and a mass of amino acids, which have remarkable roles in the pathogenesis of obesity and maintenance of energy balance. Gut microbiota-derived LPS increases the plasma concentration of LPS in metabolic diseases, which is supported by the finding that obese individuals have elevated plasma contents of LPS than lean individuals (54). Mechanically, gut microbiota-derived LPS is taken up, then trafficked to adipose tissue, where it is internalized by adipocytes and ATMs, resulting in adipose tissue expansion and the transition of macrophages with the M2 phenotype to the M1 phenotype in a manner dependent on intact TLR4 signaling (55). In addition, Hersoug et al. summarized that LPS is implicated in defining the adipocyte death size and generation of crown-like structures (56–59). Based on these previous findings, microbiota-derived LPS can signal in an endocrine manner by entering the circulation to communicate with ATMs, shedding light on the potential of a microbiota-derived LPS therapeutic approach for treating obesity.

The amount of SCFA-generating bacteria and SCFAs is diminished in fecal specimens of dysmetabolic mice (57) and in humans with obesity and diabetes (58). Ni et al. indicated that SCFAs protect against LPS-triggered dysfunction of the intestinal barrier and indirectly improve LPS-triggered ATM recruitment (59). SCAFs can also cross talk with specific cell surface receptors for instance the G-protein-coupled receptor (GPR) 43 and GPR41 (60), and can also reach circulation, thus directly affecting peripheral tissue substrate metabolism and function (61). Nakajima et al. investigated the role of GPR43 in ATMs. They suggested that SCFA-activated GPR43 plays a direct role in suppressing fat accumulation and regulating inflammatory signals in adipose tissue M2-type macrophages (62). Similar to SCFAs, gut microbiota-derived succinate is akin to hormones by engaging succinate receptor 1 (SUCNR1) (63). Circulating succinate is elevated in obesity and is a promising clinical tool for early detection of metabolic dysfunction (64, 65). More recent observations point to a succinate–SUCNR1 axis acting as an integrator of macrophage immune response in metabolic disease (65). Notably, the impact of SUCNR1 insufficiency of ATMs is dependent on their location, as well as inflammatory status. SUCNR1-insufficient ATMs from subcutaneous fat show a pro-inflammatory phenotype, whereas SUCNR1-deficient ATMs from visceral fat (which display a typical pro-inflammatory phenotype) exhibit a dampened inflammatory status (66). Intriguingly, an intraperitoneal injection of butyrate in db/db mice markedly reduced the expression of inflammatory factors in subcutaneous adipose tissue and improved obesity-triggered IR in conjunction with the decreased expression of inflammatory ATM marker genes (67). Thus, gut microbiota-derived metabolites could be a possible target for furthering our current comprehension of the relationship of the gut with ATMs.

### 3.2 Gut Cytokines

Enteroendocrine cells generate a range of gut cytokines that play key roles in metabolism, as they are associated with intestinal function, insulin secretion, and appetite (68). Among them, glucagon-like peptide-1 (GLP-1), secreted from intestinal L-cells in response to nutrient ingestion, has attracted much attention because of its mimetics for the treatment of obesity (69), lowering glucose levels, and improving insulin sensitivity in individuals with type 2 diabetes and in animal models (70). A large study population of 1462 Danish adults demonstrated that individuals who were obese or overweight had up to a 20% diminished GLP-1 response to oral glucose in contrast with normal weight individuals (71). Lee et al. initially reported that GLP-1 alleviates macrophage infiltration in the adipose tissue of leptin deficient (ob/ob) mice, which was accompanied by a reduction of M1-specific mRNAs (F4/80 and Tlr4), whereas no difference in M2 marker genes was observed through the direct inhibition of inflammatory signaling pathways (NF-κB) (72). GLP-1 receptors (GLP-1R) are abundantly expressed on the surface of numerous cell types, including pancreatic islet cells, gastrointestinal cells, neural cells, as well as mononuclear macrophages (73). It has been suggested that GLP-1/GLP-1R signaling dampens M1 activation and triggers M2 activation by cyclic adenosine monophosphate (cAMP)/protein kinase A (PKA) mediated c-Jun N-terminal kinase (JNK) downregulation in RAW264.7 cells (73). Based on these findings, we speculate that GLP-1 alleviates macrophage recruitment in adipose tissue and regulates ATMs from an M1 to M2 phenotype in an endocrine manner, thereby alleviating obesity-related inflammation.

Ghrelin, another gastrointestinal cytokines, acts *via* the growth hormone secretagogue receptor (GHSR) and is known to increase appetite and promote obesity (74, 75). Previous studies found that ghrelin levels in obese individuals are decreased (76, 77) and that GHSRs are expressed in ATMs (78). A previous study indicated that acyl ghrelin could enhance macrophage polarization to M1 directly *in vitro*, and deletion of its receptor GHSR in obese mice reduced macrophage infiltration, promoted macrophage polarization to M2 in adipose tissue, and suppressed adipose tissue inflammation (79); thus, these data suggest that Ghrelin/GHSR axis acts as an endocrine signal to induce a pro-inflammatory M1 phenotype in adipose tissue and that the deletion of GHSR promotes anti-inflammatory effects in obesity. Further investigations are needed to investigate the role of ATM-specific deletion in GHSR-induced obese mice on ATM polarization.

## 4 The Crosstalk Between Skeletal Muscle and ATMs

In recent years, considerable attention has been paid to the identification of novel functions of skeletal muscles, which release myokines and may act in an endocrine approach to facilitate tissue-to-tissue communication. Muscle contraction is a primary modulator of myokine expression and releases myokines, such as IL-6, irisin, beta-aminoisobutyric acid (BAIBA), and myonectin, exerting an effect on adipogenesis and metabolism (80–83). Importantly, skeletal muscle also releases exosomes into circulation, which can be incorporated into the distal organ and represent a new endocrine signal. Here, we summarize three candidate myokines (IL-6, FNDC5 and MSTN); we speculate that they may exert effects on the recruitment and polarization of macrophages in adipose tissue, thus regulating inflammation states in obesity.

### 4.1 Myokines

Interleukin 6 (IL-6) was the first myokine to be discovered and is the best-studied metabolic myokine (84, 85). Intramuscular IL-6 facilitates glucose uptake and fat oxidation through PI3K and AMPK signaling pathways, respectively (86). A study showed that physical activity induces muscle IL-6 production and leads to elevated circulating IL-6 levels (87). Wedell et al. demonstrated that the exercise-induced loss of visceral adipose tissue mass was dependent on IL-6 (88). Of note, IL6 is secreted by several tissues, and the role of individual cell types can induce different inflammatory signaling responses. Han et al. demonstrated that IL-6 secreted by skeletal muscle following exercise suppresses ATM accumulation by endocrine signaling, while adipocyte IL-6 promotes HFD-induced ATM accumulation by paracrine signaling (89), thereby indicating that different sources of IL-6 production have distinct ways in which they physiologically regulate metabolism.

Fibronectin type III domain-containing protein 5 (FNDC5) is another novel myokine secreted by contracting skeletal muscles, inducing some beneficial effects of exercise *via* the browning of white fat (84). Recently, Xiong et al. confirmed that FNDC5 has a considerable effect on obesity-induced inflammation and IR. They found that FNDC5 overexpression attenuates adipose tissue inflammation in obesity by inhibiting macrophage recruitment and M1 phenotype polarization *via* AMPKα signaling in HFD-induced obese mice (90). The data highlight that the anti-inflammatory effect of FNDC5 may be involved in the beneficial roles of appropriate exercise in some chronic metabolic diseases.

Myostatin (MSTN), a myokine known to inhibit skeletal muscle growth, has been associated with the development of obesity and IR (91). The serum levels of MTSN were found to be increased in obese individuals compared to lean individuals and were not significantly upregulated in adipose tissue (91). Furthermore, it has been demonstrated that the observed differences in serum levels are likely due to the overproduction of MSTN from skeletal muscle in obesity. When MSTN was blocked in mice fed HFD by injecting a peptibody, it suppressed HFD-induced infiltration of macrophages and the expression of proinflammatory cytokines in adipose tissue (92). In addition, MSTN inhibition increased irisin production in the muscle and serum and stimulated macrophage polarization from M1 to M2 types in adipose tissue, thus suppressing inflammation. Collectively, in an obese state, MSTN functions in an endocrine manner to increase ATM infiltration and indirectly elicit an M1 phenotype in ATMs by promoting irisin expression.

### 4.2 Skeletal Muscle-Derived Exosomes

Exercise-mediated contractility of skeletal muscle induces the release of exosomes into the extracellular milieu, thereby promoting metabolic systemic effects of endurance exercise (93). Compared with standard diet (SD) -fed mice, skeletal muscle releases more exosomes after HFD feeding in mice, and skeletal muscle-derived exosomes can be key communication messengers that regulate glucose uptake and metabolic homeostasis (94). Interestingly, long-term exercise-derived exosomal miR-342-5p serves as a cardio protective signal and protects against myocardial ischemia/reperfusion (MI/R) injury in rats (95). Further studies are required to demonstrate whether skeletal muscle-derived exosomes can reach the blood circulation and then regulate macrophage recruitment, the phenotypic change of ATMs, thus deciphering an exosome-based therapeutic approach for treating obesity and adipose tissue inflammation.

## 5 Crosstalk Between the Liver and ATMs

Like adipose tissue and skeletal muscle, the liver affects inflammation, IR, and whole-body energy homeostasis by releasing liver-derived proteins, such as hepatokines, into circulation (96). Several hepatokines may affect glucose and lipid metabolism, and thereby affect the risk of developing type 2 diabetes mellitus and metabolic disorders (97, 98). In addition, the liver modulates adipose tissue metabolic functions by directly secreting miRNA-containing hepatocyte-derived exosomes in the context of lipid overload (99). Here, we speculate that hepatokines (fetuin-A, RBP4) and hepatocyte-derived exosomes might drive ATM-mediated inflammation in obesity.

### 5.1 Hepatokines

Fetuin-A, also known as alpha-2-Heremans-Schmid glycoprotein, has become one of the most important hepatokine, and has been found to act as a novel player in regulating human metabolism (98, 100, 101). A recent study showed that fetuin-A serves as an adaptor protein for saturated fatty acids and subsequently activates TLR4, triggering the production of proinflammatory cytokines (101). Human clinical data reported by Stefan and Häring also support this finding (100). In addition, some of the beneficial effects of exercise on insulin sensitivity may be explained by changes in circulating fetuin-A and free fatty acids, promoting less TLR4 signaling in adipose tissue by modulating adipose tissue macrophage-related gene expression and the M1-like phenotype (102). Interestingly, a recent study found a significant up-regulation in circulating fetuin-A after 60 days of bed rest, accompanied by deteriorative insulin sensitivity which could not be ameliorated by reactive jump training, thus indicating that fetuin-A may be a possible biological and predictive marker of metabolic diseases (103). These studies suggest that fetuin-A is secreted in adipose tissue and cultured adipocytes; fetuin-A is also considered an adipokine (104–106), since it is known primarily as a classical hepatokine. Chatterjee et al. showed that lipid-induced fetuin-A from adipocytes promoted macrophages infiltration into adipose tissue and the conversion of anti-inflammatory M2 to pro-inflammatory M1 (107). Collectively, these findings suggest that fetuin-A acts as a hepatokine or an adipokine, exerting effect on ATM in an endocrine or paracrine manner.

Retinol-binding protein-4 (RBP4), another hepatokine, is elevated in the serum of obese humans (108). Recent rodent studies have shown that elevated serum RBP4 levels cause adipose tissue inflammation, at least partly, by increasing ATMs, and the expression of both M1 and M2 macrophages was elevated in adipose tissue (109, 110). Considering that RBP4 is an adipose and liver secreted protein, it can be either a hepotokine or an adipokine (111, 112), thus regulating ATM function in an endocrine or paracrine manner. However, the studies concerning crosstalk between hepatokines and ATMs are limited, and more work is needed to integrate the hepatokines and inflammation network and to fully understand the role of cytokines in metabolic dysregulation.

### 5.2 Hepatocyte-Derived Exosomes

Exosomes, as novel carriers in inter-tissue communication, play a role in liver-fat crosstalk. Wu et al. demonstrated that hepatic exosomes-derived miR-130a regulates energy metabolism in the adipose tissue of DIO mice (113). Mechanically, they identified that hepatic exosomes-miR-130a suppresses adipogenesis by inhibiting fatty acid synthase (FSAN) and peroxisome proliferator-activated receptor gamma (PPARγ) in 3T3-L1 cells, improving impaired glucose tolerance by suppressing pleckstrin homology domain leucine-rich repeat protein phosphatase 2 (PHLPP2) to activate the protein kinase B (AKT) – phosphorylation of Akt substrate of 160 kDa (AS160)–glucose transporter 4 (GLUT4) signaling pathway in adipocytes (113). Of note, it has been shown that the liver plays an early role in the etiology of metabolic disorders associated with lipid overload and then sends a signal to modulate the metabolic functions of adipose tissue by secreting miRNA-containing hepatocyte-derived EVs (99). Recently, it has been demonstrated that hepatocyte-derived exosomes from chronic obese mice stimulate the proinflammatory M1-like state of ATMs (114). Moreover, the proinflammation effects were due to exosomal miR-434-3p, which is enriched in these hepatocyte exosomes and this miRNA can directly promote macrophage polarization towards the M1-like state both *in vitro* and *in vivo* (114). Overall, these findings suggest that the liver plays a crucial, active metabolic regulatory role by directing crosstalk with adipose tissue *via* specific exosomes containing miRNAs.

## 6 Perspective: Interaction of Organokines With Organokines and Organokines With Exosomes to Regulate ATM Polarization and Recruitment

As described above, we summarized the effects of various organokines and exosomes on the recruitment and polarization of ATMs in obesity. However, far beyond understanding these organokines and exosomes alone, it is crucial to emphasize that they act together in the body, forming a complex network of actions, which mediates additional favorable interactions with other organokines or exosomes and may induce indirect effects on ATMs

### 6.1 The Interaction of Various Organokines

Obesity is accompanied by changes in the adipokine, hepatokine, myokine, gut cytokines, and gut microbiota-derived metabolites. These organokines are further participated in an intricate cross-regulation with each other and maintain a systemic inflammatory environment. For example, adiponectin (a representative adipokine) can be released by the upregulation of hormones, such as irisin, IL-15, fibroblast growth factor 21(FGF21), zinc-α2-glycoprotein, MSTN, and osteocalcin (115–118), whereas fetuin-A can inhibit the release of adiponectin. Furthermore, alteration of gut microbiota induced by antibiotic use can elevate the expression of adiponectin in HFD mice by modifying promoter DNA methylation, and supplementation of gut microbiota-derived metabolites (SCFAs, enone fatty acids) can reverse the level of adiponectin in obese mice (119–121). Thus, alterations in adiponectin levels caused by these other organokines may indirectly affect the recruitment and polarization of ATMs. Physical exercise enhances the production of myokine IL-6. In turn, it has been demonstrated to increase secretion of gut cytokines GLP-1, thus improving glucose and insulin secretion (82). Moreover, MSTN was found to promote the release of inflammatory adipokines and hepatokines while inhibiting other anti-inflammatory organokines, indicating the crosstalk among various organokines (116, 122). In a recent review, Suriano et al. suggested that the gut microbiota can modulate the profile of myokines and adipokines, and the interaction was bidirectional (123). As discussed, in obese state, the release and complex interaction of these organokines indirectly change the phenotype of ATM, further modulating chronic inflammation.

### 6.2 The interaction of Organokines and Exosomes

In recent years, accumulating evidence supports a role for extracellular vesicles in obesity-associated metabolic disturbance by carrying cargos (124, 125). In addition to organokines, exosomes can perform a “hormonal” function (126), and they may further interact and regulate each other and indirectly modulate the polarization of ATMs. For example, adiponectin was found to stimulate exosome biogenesis and secretion through binding to T-cadherin, leading to the reduction of cellular ceramides (127). While suppression of ceramide synthesis in whole-body and adipose tissue-specific increases anti-inflammatory M2 macrophages preferentially in subcutaneous white adipose tissue of obese mice, and promoted fibroblast growth factor 21(FGF21) and inhibited secretion of leptin (128). Considering that circulating exosome miRNA levels are substantially decreased in ADicerKO(adipose-tissue-specific knockout of the miRNA-processing enzyme Dicer) mice, it was believed that this may be due to the extreme reduction of adiponectin and adiponectin-mediated exosome synthesis in lipodystrophic mice (129, 130). Thus, adiponectin may be a crucial organokine altering the game of this small vesicle world. In addition, adipocyte-derived exosomal miR-99b could reduce FGF21 levels in the liver and impair hepatic insulin signaling pathways (129). Furthermore, it has been proved that 328 reported adipokines were identified in small extracellular vesicles derived from adipose tissue, whereas almost 75% of the known myokines were within exosomes/microvesicles (129). However, further analysis is required to explore the interplay between exosomes and organokines and their roles in ATMs.

## 7 Conclusion

The present review summarizes the endocrine organs release circulating triggers might crosstalk with ATMs, which are account for ATM infiltration and activation during obesity: (i) Adipose tissue, gut, skeletal muscle, and the liver can actively release secreted factors termed “organokines”, that is, adipokines, gut microbiota-derived metabolites, gut hormones, myokines, and hepatokines, respectively, which participate in macrophage recruitment and in the phenotypic change of ATMs.(ii) We address how these metabolic organs i.e. adipose tissue, skeletal muscle, and the liver secrete exosomes that may be responsible for ATM recruitment and activation. (iii) We highlight the interplay between various organokines and exosomes in obese state indirectly alter the ATMs phenotype and infiltration, further mediating chronic inflammation. Given the interaction between these triggers, it is difficult to target one organokine or exosome that regulates adipose tissue inflammation and the inflammatory phenotype of ATMs. Overall, further studies examining the relationships between various regulatory factors and ATM recruitment and polarization are likely to provide new strategies for the clinical treatment of obesity.

## Author Contributions

Y-YW: Data curation, software, visualization, writing—original draft, and writing—review and editing. Y-DW: Writing—original draft and revising manuscript. X-YQ: Data curation. Z-ZL: Data curation. Y-NM: Writing—review and editing. X-HX: Conceptualization and supervision. All authors contributed to the article and approved the submitted version.

## Funding

This systematic received funding from National Natural Science Foundation of China (82070873, 82000813) and Major special projects of Hunan provincial health and family planning commission (A2017011).

## Conflict of Interest

The authors declare that the research was conducted in the absence of any commercial or financial relationships that could be construed as a potential conflict of interest.

## Publisher’s Note

All claims expressed in this article are solely those of the authors and do not necessarily represent those of their affiliated organizations, or those of the publisher, the editors and the reviewers. Any product that may be evaluated in this article, or claim that may be made by its manufacturer, is not guaranteed or endorsed by the publisher.
