# Supplementary material for: Organokines and Exosomes: Integrators of Adipose Tissue Macrophage Polarization and Recruitment in Obesity
Source: Front Endocrinol (Lausanne). 2022 Feb 18;13:839849. doi: 10.3389/fendo.2022.839849 (PMC8902818; doi:10.3389/fendo.2022.839849)
Supplement: Supplementary file 1 [file DataSheet_1.pdf]

Supplemental table-the effect of some well-documented adipokines or lesser-known adipokines on ATMs polarisation and recruitment in obesity.

Adipokines	Chang in obesity	Impact on ATMs recruitment	Impact on ATMs polarisation
Adiponectin	Decrease [1,2]	Decrease [3]	M1↓ M2↑ [3]
Leptin	Increase [4,5]	Increase [6]	M1 ↑ M2 ↓[6,7]
LCN	Increase [8]	Increase [9]	M1↓ M2↑[10]
NOV/CCN3	Increase [11,12]	Increase [13]	M1 ↑ M2 ↓ [13]
SFRP5	Decrease [14]	Decrease [15]	M1↓ M2: uncertain[15]
SPX	Decrease [16,17]	Decrease [18]	M1↓ M2↑ [18]
MCP-1	Increase [19]	Increase [20,21]	M1 ↑ M2 ↓[21]
CXCL12	Increase [22]	Increase [23]	M1↑ M2: unchanged [23]
CXCL14	Increase [24,25]	Increase [26,27]	M1↓ M2↑ [28]
CXCL18	Increase [19]	Increase [29]	M1↑ M2↓[30]

Abbreviations: LCN2: lipocalin2, SFRP5: secreted frizzled-related protein5, SPX: spexin, MCP-1: monocyte chemoattractant protein-1, CXCL: the chemokine ligand.

Reference:

1. Arita Y, Kihara S, Ouchi N, Takahashi M, Maeda K, Miyagawa J, et al. Paradoxical decrease of an adipose-specific protein, adiponectin, in obesity. 1999. *Biochem Biophys Res Commun*. 2012;425(3):560-564. doi:10.1016/j.bbrc.2012.08.024
2. Hotta K, Funahashi T, Arita Y, Takahashi M, Matsuda M, Okamoto Y, et al. Plasma concentrations of a novel, adipose-specific protein, adiponectin, in type 2 diabetic patients. *Arterioscler Thromb Vasc Biol.* 2000;20(6):1595-1599. doi:10.1161/01.atv.20.6.1595
3. Ohashi K, Parker JL, Ouchi N, Higuchi A, Vita JA, Gokce N, et al. Adiponectin promotes macrophage polarization toward an anti-inflammatory phenotype. *J Biol Chem.* 2010;285(9):6153-6160. doi:10.1074/jbc.M109.088708
4. Monteiro L, Pereira JADS, Palhinha L, Moraes-Vieira PMM. Leptin in the regulation of the immunometabolism of adipose tissue-macrophages. *J Leukoc Biol.* 2019;106(3):703-716. doi:10.1002/JLB.MR1218-478R)
5. Considine RV, Sinha MK, Heiman ML, Kriauciunas A, Stephens TW, Nyce MR, et

al. Serum immunoreactive-leptin concentrations in normal-weight and obese humans. *N Engl J Med.* 1996;334(5):292-295. doi:10.1056/NEJM199602013340503

6. Zhao S, Li N, Zhu Y, Straub L, Zhang Z, Wang MY, et al. Partial leptin deficiency confers resistance to diet-induced obesity in mice. *Mol Metab.* 2020;37:100995. doi:10.1016/j.molmet.2020.100995
7. Chen YS, Liu HM, Lee TY. Ursodeoxycholic Acid Regulates Hepatic Energy Homeostasis and White Adipose Tissue Macrophages Polarization in Leptin-Deficiency Obese Mice. *Cells*. 2019 Mar 16;8(3):253. doi: 10.3390/cells8030253.
8. Mosialou I, Shikhel S, Luo N, Petropoulou PI, Panitsas K, Bisikirska B, et al. Lipocalin-2 counteracts metabolic dysregulation in obesity and diabetes. *J Exp Med.* 2020;217(10):e20191261. doi:10.1084/jem.20191261
9. Law IK, Xu A, Lam KS, Berger T, Mak TW, Vanhoutte PM, et al. Lipocalin-2 deficiency attenuates insulin resistance associated with aging and obesity. *Diabetes*. 2010;59(4):872-882. doi:10.2337/db09-1541
10. Guo H, Jin D, Chen X. Lipocalin 2 is a regulator of macrophage polarization and NF-κB/STAT3 pathway activation. *Mol Endocrinol.* 2014;28(10):1616-1628. doi:10.1210/me.2014-1092
11. Pakradouni J, Le Goff W, Calmel C, Antoine B, Villard E, Frisdal E, et al. Plasma NOV/CCN3 levels are closely associated with obesity in patients with metabolic disorders. *PLoS One.* 2013 Jun 13;8(6):e66788. doi: 10.1371/journal.pone.0066788.
12. Li JY, Wang YD, Qi XY, Ran L, Hong T, Yang J, et al. Serum CCN3 levels are increased in type 2 diabetes mellitus and associated with obesity, insulin resistance and inflammation. *Clin Chim Acta*. 2019;494:52-57. doi:10.1016/j.cca.2019.03.006
13. Martinerie C, Garcia M, Do TT, Antoine B, Moldes M, Dorothee G, et al. NOV/CCN3: A New Adipocytokine Involved in Obesity-Associated Insulin Resistance. *Diabetes.* 2016;65(9):2502-2515. doi:10.2337/db15-0617
14. Hu Z, Deng H, Qu H. Plasma SFRP5 levels are decreased in Chinese subjects with obesity and type 2 diabetes and negatively correlated with parameters of insulin resistance. *Diabetes Res Clin Pract.* 2013;99(3):391-395. doi:10.1016/j.diabres.2012.11.026
15. Ouchi N, Higuchi A, Ohashi K, Oshima Y, Gokce N, Shibata R, et al. Sfrp5 is an anti-inflammatory adipokine that modulates metabolic dysfunction in obesity. *Science.* 2010;329(5990):454-457. doi:10.1126/science.1188280
16. Walewski JL, Ge F, Lobdell H 4th, et al. Spexin is a novel human peptide that reduces adipocyte uptake of long chain fatty acids and causes weight loss in rodents with diet-induced obesity. *Obesity (Silver Spring).* 2014;22(7):1643-1652. doi:10.1002/oby.20725
17. Lin CY, Huang T, Zhao L, Zhong LLD, Lam WC, Fan BM, et al. Circulating Spexin Levels Negatively Correlate With Age, BMI, Fasting Glucose, and Triglycerides in Healthy Adult Women. *J Endocr Soc.* 2018;2(5):409-419. Published 2018 Apr 3. doi:10.1210/js.2018-00020
18. Gambaro SE, Zubiría MG, Giordano AP, Portales AE, Alzamendi A, Rumbo M, et al. "Spexin improves adipose tissue inflammation and macrophage recruitment in

obese mice". *Biochim Biophys Acta Mol Cell Biol Lipids*. 2020;1865(7):158700. doi:10.1016/j.bbalip.2020.158700
19. Kim CS, Park HS, Kawada T, Kim JH, Lim D, Hubbard NE, et al. Circulating levels of MCP-1 and IL-8 are elevated in human obese subjects and associated with obesity-related parameters. *Int J Obes (Lond).* 2006;30(9):1347-1355. doi:10.1038/sj.ijo.0803259
20. Amano SU, Cohen JL, Vangala P, Tencerova M, Nicoloro SM, Yawe JC, et al. Local proliferation of macrophages contributes to obesity-associated adipose tissue inflammation. Cell Metab. 2014;19(1):162-171. doi:10.1016/j.cmet.2013.11.017
21. Kamei N, Tobe K, Suzuki R, Ohsugi M, Watanabe T, Kubota N, et al. Overexpression of monocyte chemoattractant protein-1 in adipose tissues causes macrophage recruitment and insulin resistance. *J Biol Chem.* 2006;281(36):26602-26614. doi:10.1074/jbc.M601284200
22. Blogowski W, Serwin K, Budkowska M, Salata D, Dolegowska B, Lokaj M, et al. Clinical analysis of systemic and adipose tissue levels of selected hormones/adipokines and stromal-derived factor-1. *J Biol Regul Homeost Agents.* 2012;26(4):607-615.
23. Kim D, Kim J, Yoon JH, Ghim J, Yea K, Song P, et al. CXCL12 secreted from adipose tissue recruits macrophages and induces insulin resistance in mice. *Diabetologia.* 2014;57(7):1456-1465. doi:10.1007/s00125-014-3237-5
24. Weisberg SP, McCann D, Desai M, Rosenbaum M, Leibel RL, Ferrante AW Jr. Obesity is associated with macrophage accumulation in adipose tissue. *J Clin Invest.* 2003;112(12):1796-1808. doi:10.1172/JCI19246
25. Xu H, Barnes GT, Yang Q, Tan G, Yang D, Chou CJ, et al. Chronic inflammation in fat plays a crucial role in the development of obesity-related insulin resistance. *J Clin Invest.* 2003;112(12):1821-1830. doi:10.1172/JCI19451
26. Nara N, Nakayama Y, Okamoto S, Tamura H, Kiyono M, Muraoka M, et al. Disruption of CXC motif chemokine ligand-14 in mice ameliorates obesity-induced insulin resistance. *J Biol Chem.* 2007;282(42):30794-30803. doi:10.1074/jbc.M700412200
27. Ignacio RM, Gibbs CR, Lee ES, Son DS. Differential Chemokine Signature between Human Preadipocytes and Adipocytes. *Immune Netw.* 2016;16(3):189-194. doi:10.4110/in.2016.16.3.189;
28. Cereijo R, Gavaldà-Navarro A, Cairó M, Quesada-López T, Villarroya J, Morón-Ros S, et al. CXCL14, a Brown Adipokine that Mediates Brown-Fat-to-Macrophage Communication in Thermogenic Adaptation. *Cell Metab.* 2018;28(5):750-763.e6. doi:10.1016/j.cmet.2018.07.015
29. Yamaguchi R, Yamamoto T, Sakamoto A, Ishimaru Y, Narahara S, Sugiuchi H, et al. Chemokine profiles of human visceral adipocytes from cryopreserved preadipocytes: Neutrophil activation and induction of nuclear factor-kappa B repressing factor. *Life Sci.* 2015;143:225-230. doi:10.1016/j.lfs.2015.11.010
30. Cui S, Qiao L, Yu S, Men L, Li Y, Li F, et al. The antagonist of CXCR1 and CXCR2 protects db/db mice from metabolic diseases through modulating inflammation. *Am J Physiol Endocrinol Metab.* 2019;317(6):E1205-E1217.

doi:10.1152/ajpendo.00117.2019.
